# Diurnal Variations of Mouse Plasma and Hepatic Bile Acid Concentrations as well as Expression of Biosynthetic Enzymes and Transporters

**DOI:** 10.1371/journal.pone.0016683

**Published:** 2011-02-08

**Authors:** Yu-Kun Jennifer Zhang, Grace L. Guo, Curtis D. Klaassen

**Affiliations:** Department of Pharmacology, Toxicology and Therapeutics, University of Kansas Medical Center, Kansas City, Kansas, United States of America; Innsbruck Medical University, Austria

## Abstract

**Background:**

Diurnal fluctuation of bile acid (BA) concentrations in the enterohepatic system of mammals has been known for a long time. Recently, BAs have been recognized as signaling molecules beyond their well-established roles in dietary lipid absorption and cholesterol homeostasis.

**Methods and Results:**

The current study depicted diurnal variations of individual BAs detected by ultra-performance liquid chromatography/mass spectrometry (UPLC/MS) in serum and livers collected from C57BL/6 mice fed a regular chow or a chow containing cholestyramine (resin). Circadian rhythms of mRNA of vital BA-related nuclear receptors, enzymes, and transporters in livers and ilea were determined in control- and resin-fed mice, as well as in farnesoid X receptor (FXR) null mice. The circadian profiles of BAs showed enhanced bacterial dehydroxylation during the fasting phase and efficient hepatic reconjugation of BAs in the fed phase. The resin removed more than 90% of BAs with β-hydroxy groups, such as muricholic acids and ursodeoxycholic acid, from serum and livers, but did not exert as significant influence on CA and CDCA in both compartments. Both resin-fed and FXR-null mouse models indicate that BAs regulate their own biosynthesis through the FXR-regulated ileal fibroblast growth factor 15. BA flux also influences the daily mRNA levels of multiple BA transporters.

**Conclusion:**

BA concentration and composition exhibit circadian variations in mouse liver and serum, which influences the circadian rhythms of BA metabolizing genes in liver and ileum. The diurnal variations of BAs appear to serve as a signal that coordinates daily nutrient metabolism in mammals.

## Introduction

Organisms have developed 24-hr (circadian) rhythmic physiological processes to adapt to daily environmental changes. The main circadian clock is situated in the suprachiasmatic nucleus in the brain and synchronizes peripheral clocks. However, peripheral tissues, like liver, can generate oscillations independent of the suprachiasmatic nucleus [1]. A special group of clock genes, organized in feedback loops, are responsible for the circadian rhythms in both the suprachiasmatic nucleus and peripheral organs. The phase of the suprachiasmatic nucleus neurons is mainly entrained by the light-dark cycle, whereas that of peripheral organs, like liver and intestine, is more influenced by the feeding-fasting cycles [2]. Recently, clocks in peripheral tissues, such as lung, were shown to be sensitive to temperature changes within physiological range [3].

In the enterohepatic system, bile acids (BAs) play important roles in eliminating cholesterol and solubilizing lipid nutrients [4]. Recently, BAs have been recognized as signaling molecules that orchestrate the metabolism of BAs, glucose, fatty acid, and lipoprotein synthesis through activating the nuclear receptor, farnesoid X receptor (FXR) [5], and energy expenditure via a membrane receptor, the G-protein-coupled receptor TGR5 [6]. Therefore, the diurnal variation of BA concentrations in livers and serum may influence the daily energy homeostasis in mammals.

BAs undergo extensive recycling between liver and intestine, which is termed the enterohepatic circulation (EHC) of BAs [4]. The EHC has two BA inputs, the biosynthesis of primary BAs from cholesterol or oxysterols in the liver, and the formation of secondary BAs from primary BAs by intestinal bacteria. Primary BAs in humans are chenodeoxycholic acid (CDCA; 3α,7α-dihydroxy) and cholic acid (CA; 3α,7α,12α-trihydroxy). In mice, the CDCA-derived alpha-muricholic acid (αMCA; 3α,6β,7α-trihydroxy) and beta-muricholic acid (βMCA; 3α,6β,7β-trihydroxy) [7], as well as CA are the predominant primary BAs. BAs are mainly conjugated with taurine in mice before being excreted into bile. Primary BAs that enter the intestinal lumen are efficiently reabsorbed by the small intestine. A portion of the primary BAs are deconjugated by bacteria in the distal ileum and transformed into secondary BAs in the large intestine. Bacteria convert CA into deoxycholic acid (DCA, 3α,12α-dihydroxy); CDCA into lithocholic acid (LCA, 3α-monohydroxy) and ursodeoxycholic acid (UDCA, 3α,7β-dihydroxy); αMCA into hyocholic acid (HCA, 3α,6α,7α-trihydroxy) and murideoxycholic acid (MDCA, 3α,6β-dihydroxy); and βMCA into omega-muricholic acid (ωMCA, 3α,6α,7β-trihydroxy) and hyodeoxycholic acid (HDCA, 3α,6α-dihydroxy) [4], [8].

**Figure 1:**
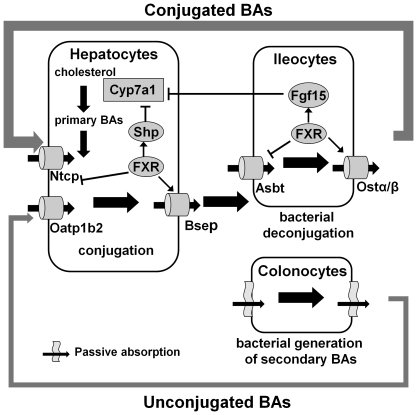
Scheme of major components of the enterohepatic circulation (EHC) of bile acids (BAs) in mice. Primary BAs are synthesized and conjugated by the liver and released into the intestine lumen to aid nutrient absorption. The majority of BAs are reabsorbed by intestinal transporters Asbt (apical) and Ostα/β (basolateral) into portal venous blood. A small portion of BAs are deconjugated by bacteria in distal ileum and further dehydroxylated or epimerized by bacteria in the large intestine to generate secondary BAs. Deconjugated primary and secondary BAs can be passively diffused into the portal blood. Ntcp is the major transporter in the liver that removes the majority of BAs from portal blood. Unconjugated BAs are taken up by organic anion transporting polypeptide 1b2 (Oatp1b2) on the basolateral membrane of hepatocytes or passive absorption. Upon entering hepatocytes, unconjugated and secondary BAs are reconjugated and rehydroxylated before they are pumped into the bile by Bsep to start another EHC. Under normal conditions, BAs are synthesized in pericentral hepatocytes, whereas BAs are taken up by periportal hepatocytes. Nuclear receptor FXR coordinates BA homeostasis by promoting (arrow) or repressing (blunt arrow) of key BA synthesis enzymes or transporters in liver and ileum. Bold arrows indicate the BA flows.

Membrane transporters in liver and ileum ensure efficient EHC of BAs (Fig. 1). In ileum, the apical sodium-dependent BA transporter (Asbt) [9] and the basolateral heterodimeric organic solute transporters alpha and beta (Ostα/Ostβ) [10] transport most of the conjugated BAs from the intestinal lumen into the portal vein. Unconjugated mono- and di-hydroxy BAs can passively diffuse through enterocytes [4]. Hepatocytes remove conjugated BAs from the portal venous blood by the sodium taurocholate cotransporting polypeptide (Ntcp) [11] and unconjugated BAs by basolateral transporters [12] such as organic anion transporting peptide 1b2 [13] or passive diffusion. BAs are excreted into canalicular bile via the ATP-energized bile salt export pump (Bsep) and multidrug-resistant protein 2 (Mrp2) [12]. Under normal physiological conditions, the EHC of BAs is so efficient that the newly synthesized BAs contribute less than 5% of the circulating BAs in humans [4].

The circadian clock is known to regulate the expression of several genes involved in BA metabolism, such as cytochrome P450 7a1 (Cyp7a1) [14], [15], the rate-limiting enzyme for BA synthesis. Interestingly, the expression of Cyp7a1 is also suppressed by FXR, which can be activated by BAs at physiological concentrations [16], [17]. Meanwhile, FXR regulates the expression of multiple BA transporters [11], [18], [19], [20]. Therefore, the food-induced BA flux provides a diurnal activation of FXR, which could, in turn, exert circadian regulations on its target genes.

The current study was designed to determine circadian rhythms of individual BAs and the influence of BAs on the daily expression of genes involved in BA metabolism in mice. To address these issues, mice were fed cholestyramine, a polymeric BA-binding resin, to interrupt the EHC of BAs, as well as FXR-null mice were used to determine the regulatory roles of FXR on enterohepatic gene expression. Insights into BA metabolism and roles of BA-FXR signaling in generating the enterohepatic circadian rhythm were obtained.

## Materials and Methods

### Ethics statement

Mice were housed according to guidelines of the Institutional Animal Care and Use Committee at the University of Kansas Medical Center, and procedures were carried out in compliance with standards for use of laboratory animals. Animal experiments performed in this manuscript have been approved by the Institutional Animal Care and Use Committee at the University of Kansas Medical Center (protocols 2005–1504 and 2008–1725).

### Animals and treatments

Male C57BL/6 mice (7 weeks of age) were purchased from Charles River (Wilmington, MA). Age-matched FXR-null mice on C57BL/6 background were bred in the animal facility at this university. All mice were acclimated in a temperature/humidity controlled facility with a 12-hr lighting schedule (05:00–17:00) for one week, and fed either regular pellet rodent chow (Harlan Teklad, Madison, WI) or ground chow supplemented with 2% (w/w) cholestyramine (Sigma, St. Louis, MO) for another week. All mice had *ad libitum* access to drinking water and feed. For resin-fed groups, diet and cages were replaced daily. Serum, livers, and ilea were collected from mice (n = 5/time point for wild-type and n = 4/time point for FXR-null) at 4-hr intervals over 24 hr. The first time point was 1 hr after the light was on. Livers and ilea were rinsed with saline, frozen in liquid nitrogen, and stored at −80°C until use.

### Quantification of BAs in mouse serum and liver

BA concentrations in mouse liver and serum were quantified by reverse-phase ultra-performance liquid chromatography-mass spectrometry (UPLC-MS/MS). Detailed preparations of serum and liver BA samples as well as internal standards were described previously [21].

### RNA extraction

Total liver and ileum RNA was prepared with RNA-Bee reagent (Tel-Test Inc., Friendswood, TX) from frozen tissues following the manufacturer's instructions. RNA concentrations were quantified with a Nanodrop nd-1000 spectrophotometer (Thermo Scientific, Wilmington, DE) and diluted to a final concentration of 1 µg/µl. Purified RNA was stored at −80°C. Five (wild-type) or 4 (FXR-null) individual samples from each group were used for gene expression assays.

### Messenger RNA quantification

Messenger RNA of target genes were quantified using branched DNA signal amplification assays (Affymetrix/Panomics, Inc., Fremont, CA) as reported previously [22]. The bDNA probe sets have been published [22], [23], [24], [25]. Ten µg of RNA was used for the assay and luminescence was quantified with a Synergy 2 Multi-Detection Microplate Reader interfaced with Gen5 Reader Control and Data Analysis Software (Biotek, Winoosky, VT). Luminescence was reported as relative light units (RLU) per 10 µg of total RNA.

### Statistical analysis

Data are presented as mean ± standard error of the mean (n = 5 or 4). Statistical differences between groups at each time point were analyzed using a student t-test (two tailed) and significance level was set at p<0.05.

## Results

### Circadian rhythms of serum BAs in control- and resin-fed mice

**Figure 2:**
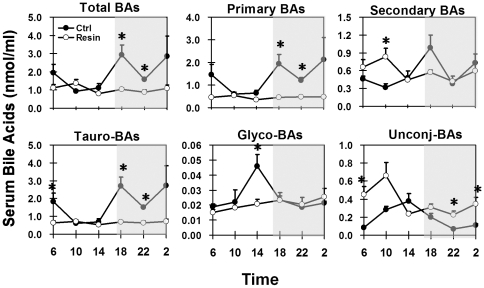
Circadian fluctuations of serum total BAs, as well as secondary transformation and conjugations in control-fed (Ctrl) and cholestyramine-fed (Resin) mice. Tauro-BAs: tauro-conjugated BAs; Glyco-BAs: glycine-conjugated BAs; Unconj-BAs: unconjugated BAs. Shaded area of the figure represents dark phase and opened area stands for light phase. Data are presented as mean ± standard error (n = 5). Asterisk stands for statistically difference between control and resin groups. p≤0.05, student t-test (two-tailed). Same illustrations apply to the following figures.

In control mice, total serum BA concentrations were lowest in the middle of the light phase (0.9 nmol/ml at 10:00), and highest in the beginning and end of the dark phase (2.9 nmol/ml at 18:00 and 02:00) (Fig. 2). About 75% of serum BAs are primary BAs, which exhibited a similar circadian pattern as total BAs. Secondary BAs had a pronounced circadian peak at 18:00. The major serum BAs were taurine-conjugated BAs (more than 93% in the dark phase), followed by unconjugated BAs. Glycine-conjugated BAs were minor components (0.7 to 4.1% during the day). Serum contained the highest percentage of unconjugated BAs at 14:00 (33.8%) and the lowest at 02:00 (3.8%).

Consuming the resin-containing diet resulted in flattened diurnal peaks of total, primary, tauro-conjugated, and glyco-conjugated BAs in mouse serum, but increased concentrations of secondary and unconjugated BAs (Fig. 2). Peak serum concentrations of secondary BAs and unconjugated BAs shifted from 18:00 and 14:00 in control mice, respectively, to 10:00 in mice fed the resin diet.

**Figure 3:**
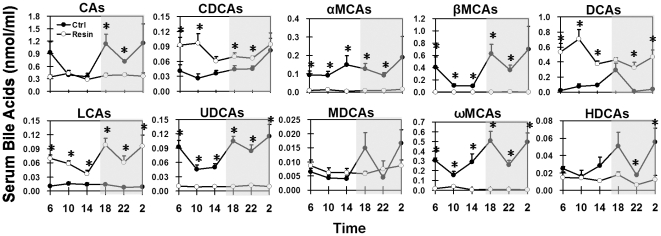
Diurnal fluctuations of individual BAs in mouse serum under control- or resin-fed conditions. Each individual BA includes both taurine-conjugated and unconjugated forms.

Individual BAs exhibited distinct circadian rhythms in serum of mice fed a control diet (Fig. 3). CA (41%) and βMCA (26%) were predominant primary BAs in mouse serum, and both exhibited similar circadian variations as that of total serum BAs described above. CDCA concentrations in mouse serum were low during most of the day and highest at 02:00. Concentrations of αMCA had two small peaks at 14:00 and 02:00.

Under control feeding conditions, DCA and ωMCA were the major secondary BAs in mouse serum (Fig. 3). DCA, which was 1.2 to 10% of the total serum BAs, had a concentration peak at 18:00. LCA concentrations were very low but somewhat higher during the late-light phase (10:00–18:00). Concentrations of UDCA, which was about 4.5% of total serum BAs, were higher in the dark phase and lower in the late-light phase. Serum concentrations of MDCA were also very low, with the highest concentrations at 18:00 and 02:00. ωMCA, the most abundant secondary BA in mouse serum, exhibited two identical peaks at 18:00 and 02:00, and a major trough at 10:00. HDCA had similar circadian peak-and-trough times as did ωMCA, but at much lower concentrations.

**Figure 4:**
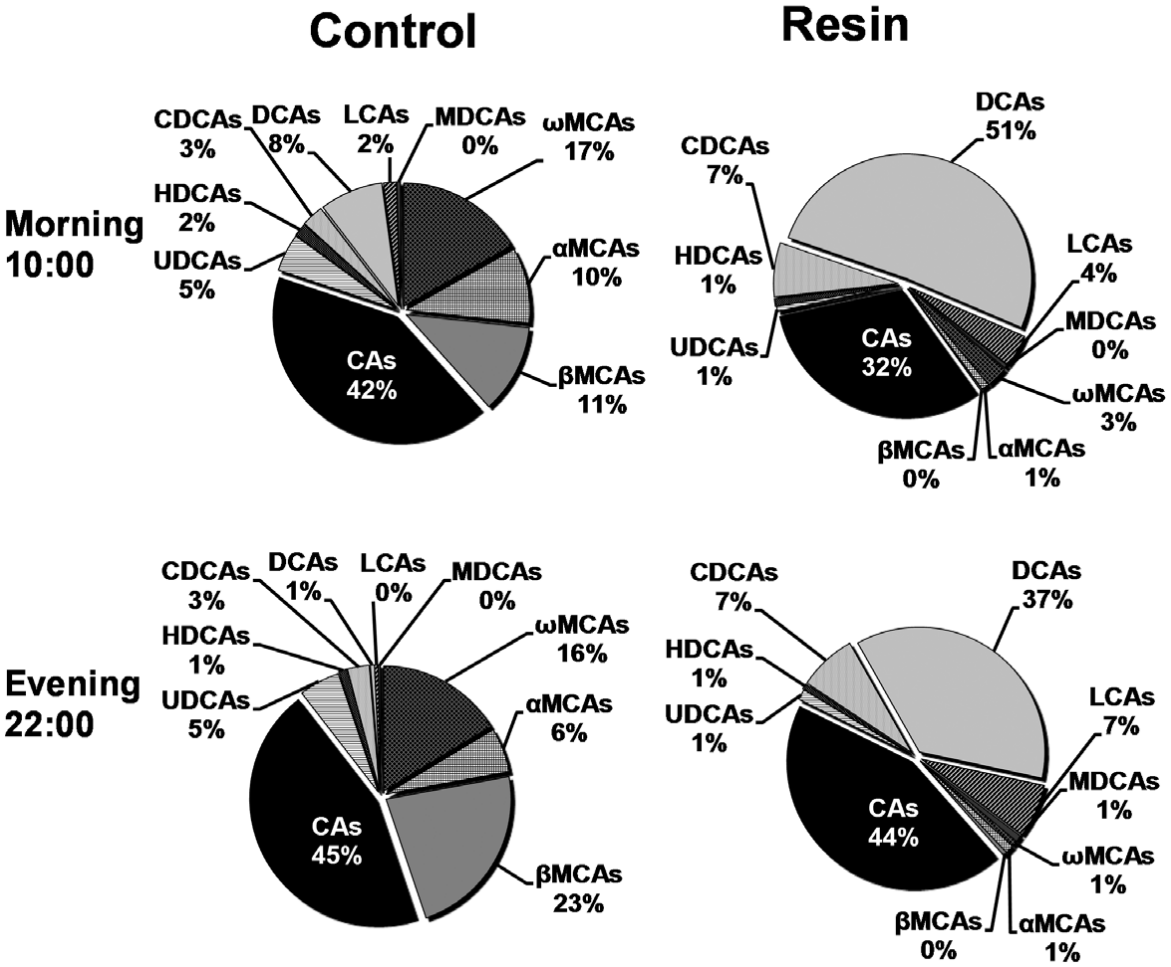
Changes of BA composition in mouse serum by cholestyramine in the morning (10:00) and evening (22:00). Each individual BA includes both taurine-conjugated and unconjugated forms.

The resin diet decreased the concentrations of αMCA, βMCA, ωMCA, and UDCA by 92, 99, 96, and 88% respectively, in mouse serum (Fig. 3). MCAs (α, β, ω) were reduced the most in resin-fed mice during both fasting (10:00) and feeding (22:00) phases. The contribution of MCAs to the total serum BAs decreased from 39% in control-fed mice to 4% in resin-fed mice at 10:00 (Fig. 4). The circadian variations of CA, MDCA, and HDCA in mouse serum were flattened by the resin diet. In contrast, the resin diet increased the daily average concentration of DCA 433%, with a pronounced peak at 10:00; CDCA 75%, with higher concentrations from 02:00 to 10:00; and LCA 494%, with two identical peaks at 18:00 and 02:00. The resin diet increased DCA concentration the most (8.2 fold at 10:00 and 37.4 fold at 22:00), and its percentage of serum total BAs increased from 8% in control mice to 51% in resin-fed mice at 10:00 (Fig. 4).

### Circadian rhythms of liver BAs in control- and resin-fed mice

**Figure 5:**
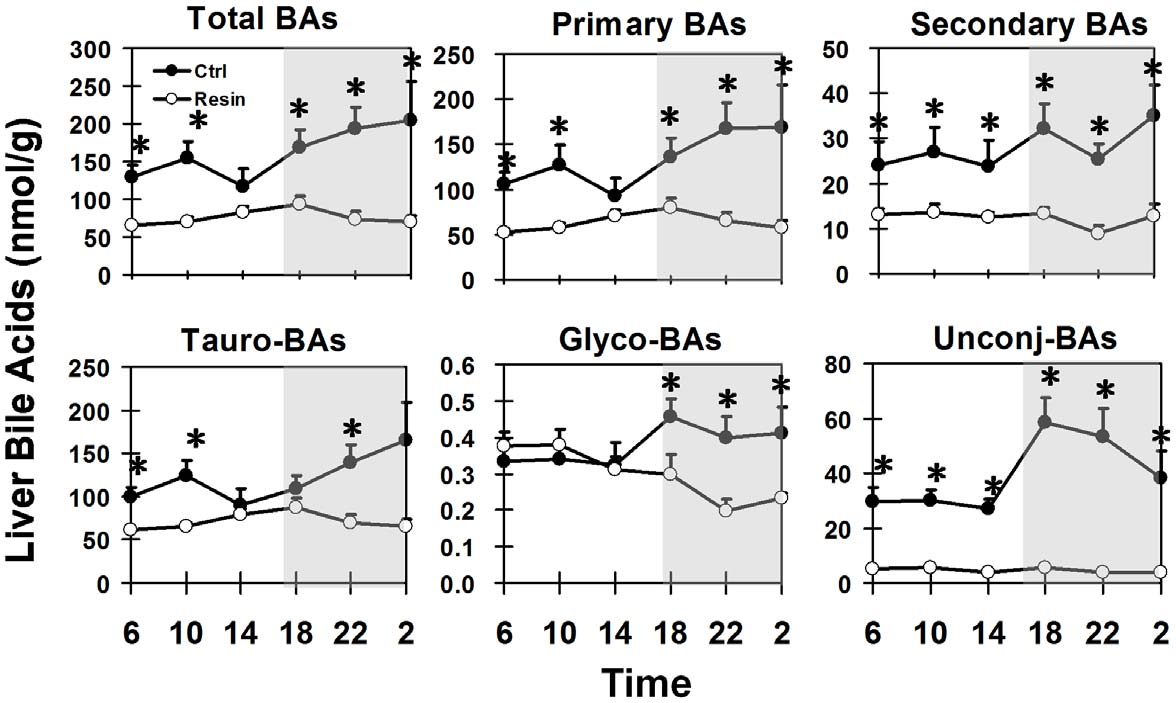
Circadian fluctuations of liver total BAs, as well as secondary transformation and conjugations in control- and resin-fed mice.

Liver BA concentrations in control-fed mice were higher in the dark/feeding phase (Fig. 5). The highest BA concentration in liver was 204 nmol/g at 02:00, and the lowest was 117 nmol/g at 14:00. Primary BAs, 79 to 87% of total BAs in the liver, exhibited similar daily fluctuations as the total BAs. Concentrations of secondary BAs, ranged from 24 to 35 nmol/g liver, did not show a significant circadian pattern. About 75% of the BAs in mouse liver were conjugated with taurine, approximately 24% unconjugated, whereas only about 0.2% was conjugated with glycine. The concentrations of conjugated BAs were higher in the dark phase, whereas unconjugated BAs had a pronounced circadian peak at 18:00, and then gradually decreased during the remaining dark phase.

Circadian peaks of BAs in mouse livers were flattened by the resin diet (Fig. 5). In livers of resin-fed mice, the daily average total BA concentration was 76 nmol/g, which was 53% less than in the control diet-fed mice. The daily average concentrations of primary, secondary, tauro-conjugated, glycine-conjugated, and unconjugated BAs in resin-fed mouse livers were 52, 55, 41, 21, and 88%, respectively, less than those in control diet-fed mice.

**Figure 6:**
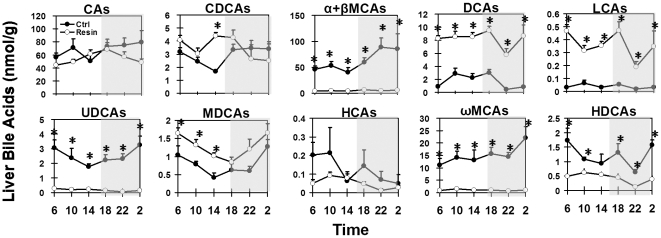
Diurnal fluctuations of individual BAs in mouse liver under control- or resin-fed conditions. Each individual BA includes both taurine-conjugated and unconjugated forms.

Individual BAs in livers of mice on the control diet exhibited diurnal fluctuations (Fig. 6). In liver samples, concentrations of αMCA and βMCA were shown as a combination (α-βMCAs) because their peaks on UPLC-MS/MS chromatograms merged. Primary BAs, including CA (51–80 nmol/g), CDCA (1.7–3.5 nmol/g), and α-βMCAs (40–89 nmol/g) were higher during the dark phase and lowest at the end of the light phase (14:00).

Similar to that in serum, ωMCA and DCA were the predominant secondary BAs in mouse livers. DCA concentrations were high from 10:00 to 18:00, and low during the rest of the dark phase. LCA concentrations were very low in mouse livers. The hepatic concentrations of UDCA varied from 1.8 (14:00) to 3.3 nmol/g (02:00). MDCA exhibited similar circadian variations as UDCA, with the highest concentration (∼1.2 nmol/g) during the late-dark phase. HCA concentration was low in livers, but was relatively high during the early-light phase. The daily average concentration of ωMCA was 15.1 nmol/g liver, with the highest at 02:00. HDCA concentration was lowest at 22:00, and highest at 02:00 and 06:00.

Feeding the resin diet decreased more than 91% of MCAs (α, β, and ω) and UDCA, 59% of HCA, and 64% HDCA, but increased DCA (376%), LCA (813%), and MDCA (62%) in mouse livers (Fig. 6). Comparisons were made on the average daily concentration of each BA. The concentrations of DCA and LCA were highest at 18:00 and lowest at 22:00. The daily average concentration of CA and CDCA was similar in livers of control- and resin-fed mice, except their circadian patterns were slightly changed. MDCA had similar circadian patterns in the two groups of mice, but at higher concentrations in livers of resin-fed mice. Concentrations of HCA and HDCA were both highest at 10:00 and lowest at 22:00 in livers of resin-fed mice.

**Figure 7:**
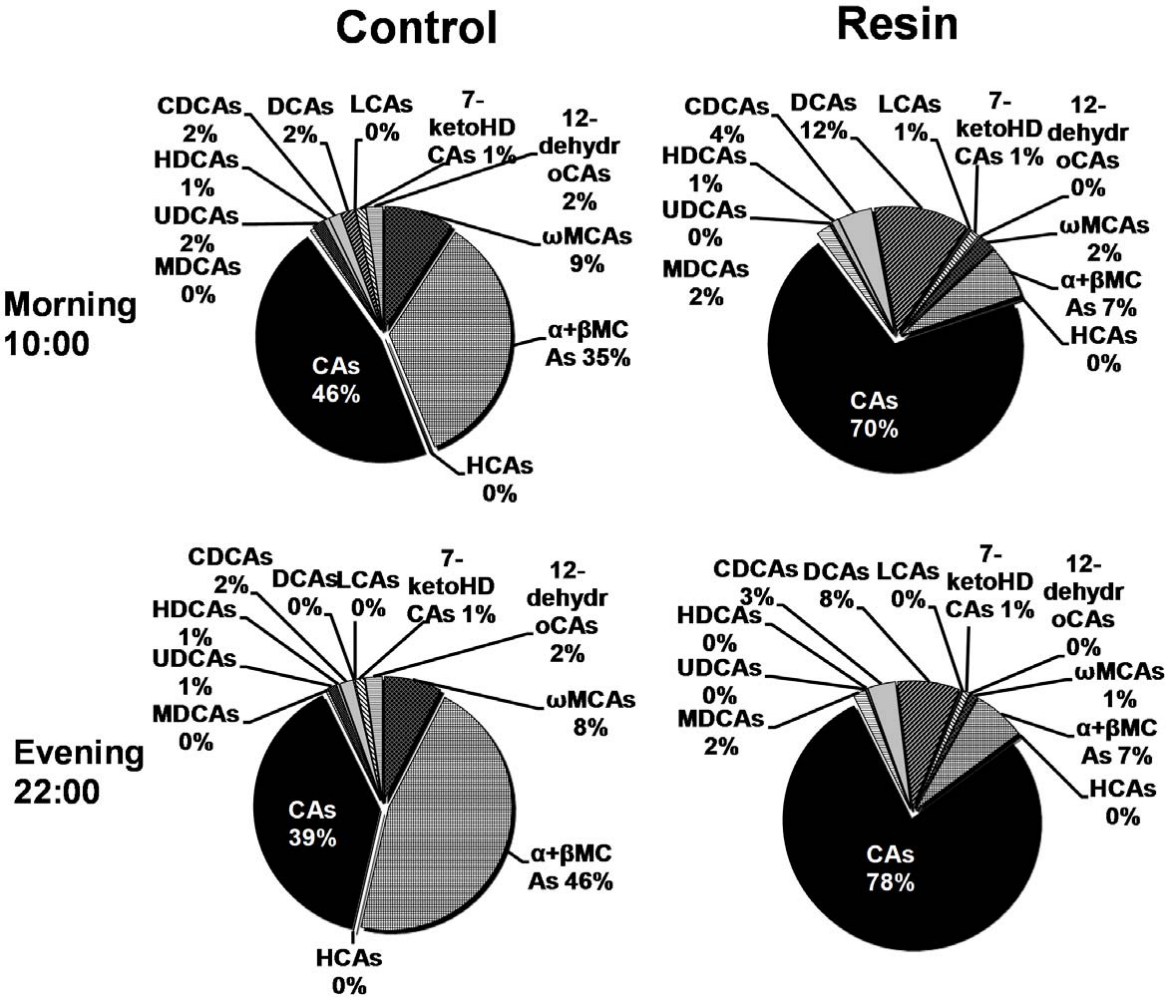
Changes of BA composition in mouse serum by cholestyramine in the morning (10:00) and evening (22:00). Each individual BA includes both taurine-conjugated and unconjugated forms.

The resin diet altered BA profiles in livers of mice during fasting (10:00) and feeding (22:00) phases (Fig. 7). The dramatic decrease of MCAs by the resin-containing diet resulted in CA being the only predominant primary BA, whereas the CA-derived DCA was the major secondary BA in livers of resin-fed mice.

### Effects of resin on daily mRNA profiles of genes encoding key proteins involved in the EHC of BAs

**Figure 8:**
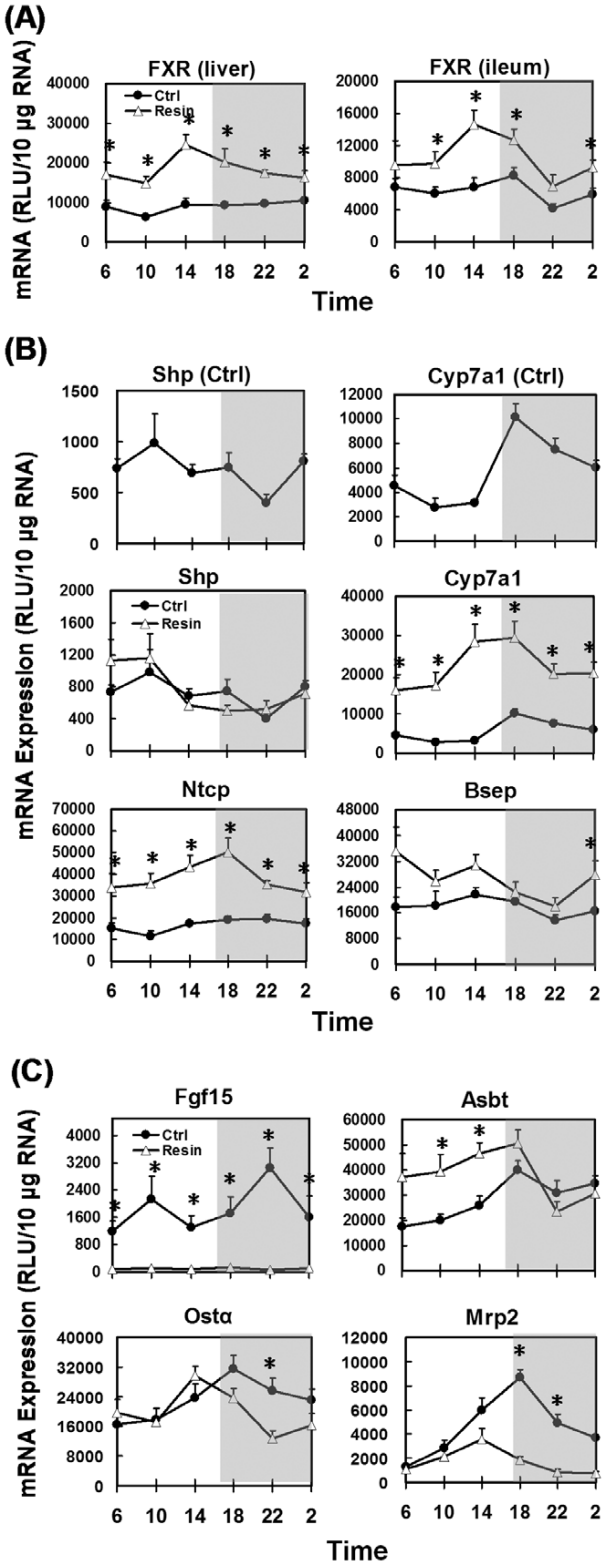
Circadian expression of hepatic and ileal genes in control- and resin-fed mice. (A) Circadian expression profiles of FXR in liver and Ileum, (B) hepatic genes, and (C) ileal genes under control- and resin-fed conditions.

In control diet-fed mice, FXR mRNA was evenly expressed throughout the 24-hr time course in both livers and ilea (Fig. 8A). The resin diet doubled the FXR mRNA content in liver, and increased it 65% in ileum. Furthermore, a pronounced circadian pattern of FXR mRNA was shown in both liver and ileum, with a peak at 14:00.

In livers of control-fed mice, Shp mRNA was highest at 10:00 in the morning and lowest at 22:00 in the evening (Fig. 8B). Cyp7a1 mRNA increased rapidly to the highest level after the onset of the dark phase (18:00), and then gradually decreased to the lowest level at 10:00. The resin diet did not influence the expression of Shp, but increased the daily average of Cyp7a1 mRNA 285%, without changing its circadian pattern. In control mice, mRNA of the BA-uptake transporter, Ntcp, was relatively lower in the early morning, and mRNA of the efflux transporter, Bsep, was slightly lower in the late evening. But neither transporters exhibited significant diurnal variations. Consumption of the resin diet increased mRNAs of Ntcp (130% daily average) and Bsep (49% daily average) in mouse livers.

In ilea (Fig. 8C), mRNA of fibroblast growth factor 15 (Fgf15) exhibited pronounced circadian rhythm with a major peak at 22:00 and a minor peak at 10:00. The resin diet reduced Fgf15 mRNA by 95% and eliminated its diurnal fluctuations. Messenger RNA of the ileal BA-uptake transporter, Asbt, reached its highest level at 18:00 and decreased to the lowest at 06:00 in regular-fed mice. The resin diet increased Asbt mRNA during the light phase. The mRNA of Ostα, half of the ileal BA basolateral efflux heterodimer, had a single peak at 18:00 in control mice. In mice fed the resin diet, Ostα mRNA levels were comparable to control mice, but had a peak at 14:00. The apical efflux transporter Mrp2 showed a marked diurnal mRNA peak at 18:00 in control mice, and the resin diet decreased it 62.5%.

### Circadian expression of key BA metabolism genes in FXR-null mice

**Figure 9:**
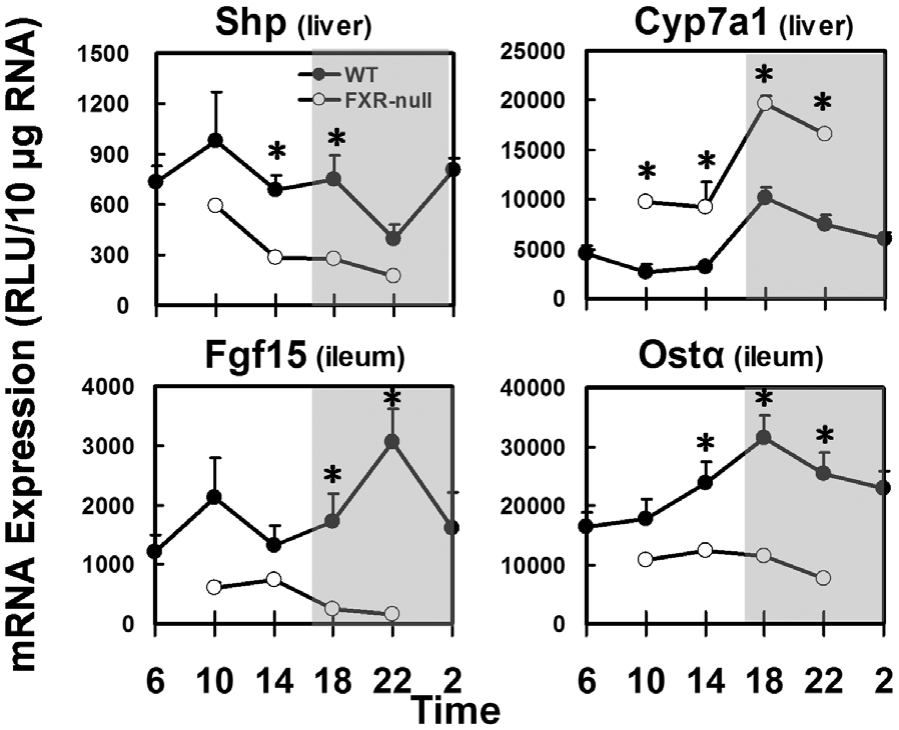
Comparing mRNAs of key hepatic and ileal BA-metabolism genes from 10:00 to 22:00 in wild-type (WT; n = 5) and FXR-null (n = 4) mice. Asterisk stands for statistically difference between wild-type and FXR-null.

To further clarify the role of FXR in regulating the circadian rhythm of BA-related genes, livers and ilea were collected from FXR-null mice from 10:00 to 22:00. In livers, FXR-null mice had 53% less Shp mRNA and 135% more Cyp7a1 mRNA than did WT mice, but the circadian patterns of both genes remained the same (Fig. 9). In ilea of FXR-null mice, the daily levels of Fgf15 mRNA and Ostα mRNA were 79 and 57% lower than in WT mice, respectively. The circadian rhythms of Fgf15 and Ostα observed in WT mice disappeared in FXR-null mice.

## Discussion

The present study depicts the circadian rhythm of BA composition during a 24-hr feeding/fasting cycle in mice. This enables us to speculate about potential metabolic pathways for BAs and discuss the influence of BAs, through their sensor FXR, on the circadian rhythm of enterohepatic genes encoding key proteins involved in BA biosynthesis and transport.

Diurnal variation of BA concentrations in serum and liver are in phase with the fasting/feeding cycle of mice. The total BA concentrations in liver and serum are lowest towards the end of the light/fasting phase (Figs. 2 and 4) when BAs are stored in the gallbladder and intestinal lumen. When mice start eating, more BAs enter the intestine lumen and intestinal absorption of BAs is accelerated, leading to increased BA concentrations in serum and liver. The two BA peaks in serum suggest that mice have two episodes of food ingestion in the dark phase, mimicking the postprandial increase of serum BAs in humans [26]. The gradual increase of liver BA concentrations during the dark phase could be the result of rapid EHC and active BA biosynthesis.

The conjugation/deconjugation of BAs exhibits a diurnal rhythm in mice. There is an increase in unconjugated BAs in serum during the light phase (Fig. 2), suggesting active deconjugation of BAs by the intestinal bacteria when mice are fasting. Livers receive the highest amount of unconjugated BAs at the beginning of the active/dark phase (Fig. 5), and efficiently reconjugate them with taurine. By the end of the dark phase, unconjugated BA concentrations decrease to low levels in both serum and liver.

The conversion between primary and secondary BAs are catalyzed by enzymes synthesized by intestinal bacteria or enzymes in the liver [4]. It is interesting that the diurnal rhythm of DCA in mouse serum is divergent from its precursor CA, whereas the circadian fluctuation of ωMCA is in phase with its precursor βMCA. One potential explanation is that bacteria catalyzing dehydroxylation (CA into DCA, and CDCA into LCA) and epimerization (βMCA into ωMCA, and CDCA into UDCA) of BAs have different activity, population size, and localization in the intestinal tract. They might have different reactions to food ingestion. It could also be because hepatic enzymes that re-hydroxylate or epimerize secondary BAs back into primary BAs have different activities during the 24-hr cycle. Another possibility is that intestinal transporters like Asbt absorb CA more efficiently than βMCA during food digestion, which protects CA from the bacterial transformation. However, these hypotheses need to be tested before firm conclusions can be made.

The circadian rhythm of BAs has been long recognized [27], [28], [29]. However, the circadian rhythm of BA metabolizing genes has not been thoroughly investigated. FXR is the key factor that regulates a broad range of genes encoding BA enzymes and transporters in liver and ileum (Fig. 1). In control-fed mice, FXR mRNA did not have a predominant diurnal rhythm in either organ. However, the resin diet markedly increased FXR mRNA and produced a circadian peak in both liver and ileum, indicating FXR might be negatively regulated by BAs.

Although both circadian clock and BA-FXR signaling are well established in regulating Cyp7a1 transcription, little is known about the influence of diurnal variation of BAs on Cyp7a1 expression. FXR negatively regulates the transcription of Cyp7a1 via Shp in liver [30] and Fgf15 from ileum [31]. The circadian rhythm of Shp mRNA is regulated by the clock gene Rev-erbα [15], and is not changed by either resin diet or FXR deficiency in mouse livers (Figs. 6 and 7). Fgf15 mRNA peaks 4-hr after that of Cyp7a1 (Fig. 8), suggesting Fgf15 contributes to the decline of liver BA biosynthesis in the late-dark phase. Similar postprandial peaks were previously observed in circulating Fgf19, the human homolog of Fgf15 [32]. The circadian expression of Fgf15 appears to depend on the activation of intestinal FXR by BAs. In resin-fed or FXR-null mice, the diminished Fgf15 signal releases the repression on Cyp7a1, leading to higher transcription of Cyp7a1 in livers. However, the circadian rhythm of Cyp7a1 is not changed in either model, suggesting the rhythmic regulation from the clock genes [14], [15]. Moreover, the FXR-dependent regulation of Fgf15 was also demonstrated in FXR ileum-specific null mice [33]. Taken together, BAs regulate Cyp7a1 mRNA through the ileal FXR-Fgf15 signaling pathway, but the circadian rhythm of Cyp7a1 is determined by clock genes.

In mice, the hepatic enzymes that convert CDCA into α- and β-MCA remain unknown. Compared to the relatively consistent concentration of CA, concentrations of αMCA and βMCA exhibit more profound diurnal fluctuations in mouse livers. Because Cyp7a1 initiates the biosynthesis of both CDCA and CA, the activity of enzymes transforming CDCA into α- and β-MCA is likely to have circadian variations.

Enhanced ileal transport upon food ingestion facilitates reabsorption of BAs from the intestine lumen back into serum and liver. This is supported by the increased mRNAs of ileal BA uptake transporter Asbt and efflux transporter Ostα at the beginning of the dark phase (Fig. 8).

FXR has been reported to regulate multiple BA transporters. In mice, the activation of FXR indirectly represses the expression of Ntcp in liver [11] and Asbt in ileum [18], and directly promotes the expression of Bsep in hepatocytes [19] and Ostα/β in ileocytes [20]. When cholestyramine is fed, the BA flux is decreased, leading to diminished activation of FXR in both liver and ileum. Accordingly, more Asbt is expressed in ileum to enhance the absorption of BAs in the ileum. Meanwhile, hepatocytes express more Ntcp to enhance the extraction of BAs from portal blood. Ostα mRNA is not decreased by BA deprivation, but is ablated in FXR-null mice, suggesting FXR regulates Ostα expression independent of BAs. Changed circadian expression profiles of Ntcp and Bsep were previously reported in mice either on a 2% cholestyramine diet or deficient in FXR under restricted feeding [34]. Mrp2 locates on the apical membrane of enterocytes and effluxes organic anions back into the intestinal lumen [35]. The transcription of Mrp2 is positively regulated by FXR [36]. The sequestration of BAs also reduced the mRNA expression of Mrp2 in ileum. Hence, BAs may diurnally influence the transcription of enterohepatic transporters through FXR.

The primary BAs decreased the most in mice by cholestyramine were α- and β-MCA, with less of a decrease of CA. Similarly, humans treated with another BA binding resin, colesevelam, had decreased CDCA but increased CA pool sizes [37]. The synthesis of conjugated CDCA and the subsequent transformation into MCAs were shown to be increased in hepatocytes isolated from rats fed cholestyramine [38]. Therefore, the removal of MCAs from the enterohepatic system of mice (CDCA in humans), which in turn promotes the biosynthesis of BAs from cholesterol in the liver, appears to be the major mechanism for the cholesterol-lowering effect of BA resins.

Omega MCA is a major secondary BA in mice. Both the current study and a previous study [21] observed markedly reduced concentrations of ωMCA in mouse livers after cholestyramine treatment. DCA concentration is increased in the resin-treated mice in the current study, but was unchanged in the previous report [21]. Taken together, cholestyramine decreased BAs having β-hydroxy groups, such as αMCA (6β-OH), βMCA (7β-OH), ωMCA (7β-OH), and UDCA (7β-OH), more than other BAs in mice (Fig. 3 and 6). Direct binding assays are required to determine whether this is due to differences in binding affinities. In addition to hepatic BA biosynthesis and cholestyramine-binding affinity, changes of intestinal microflora can also influence the bile acid profile in cholestyramine-fed mice.

In summary, the EHC of BAs has a predominant circadian rhythm that is in phase with the feeding/fasting cycle of mice. The diurnal variations of BA concentrations and composition could play important roles in coordinating nutrient absorption and energy homeostasis. FXR, in respond to daily BA fluctuations upon food ingestion, determines the basal and circadian expression of Fgf15, and regulates vital enzymes and transporters in mouse liver and ileum to maintain BA homeostasis.
